# Evidence of fNIRS-Based Prefrontal Cortex Hypoactivity in Obesity and Binge-Eating Disorder

**DOI:** 10.3390/brainsci11010019

**Published:** 2020-12-26

**Authors:** Sarah A. Rösch, Ricarda Schmidt, Michael Lührs, Ann-Christine Ehlis, Swen Hesse, Anja Hilbert

**Affiliations:** 1 Integrated Research and Treatment Center Adiposity Diseases, Behavioural Medicine Research Unit, Department of Psychosomatic Medicine and Psychotherapy, University of Leipzig Medical Center, Semmelweisstrasse 10, 04103 Leipzig, Germany; ricarda.schmidt@medizin.uni-leipzig.de (R.S.); anja.hilbert@medizin.uni-leipzig.de (A.H.); 2 International Max Planck Research School NeuroCom, Max Planck Institute for Human Cognitive and Brain Sciences, Stephanstrasse 1a, 04103 Leipzig, Germany; 3 Brain Innovation B.V., Oxfordlaan 55, 6229 EV Maastricht, The Netherlands; luehrs@brainvoyager.com; 4 Faculty of Psychology and Neuroscience, Department of Cognitive Neuroscience, Maastricht University, Oxfordlaan 55, 6229 EV Maastricht, The Netherlands; 5 Department of Psychiatry and Psychotherapy, University of Tübingen, Calwerstrasse 14, 72076 Tübingen, Germany; ann-christine.ehlis@med.uni-tuebingen.de; 6 Department of Nuclear Medicine, University of Leipzig Medical Center, Liebigstrasse 18, 04103 Leipzig, Germany; swen.hesse@uniklinik-leipzig.de

**Keywords:** obesity, binge-eating disorder, fNIRS, emotional dysregulation, prefrontal cortex, impulsivity

## Abstract

Obesity (OB) and associated binge-eating disorder (BED) show increased impulsivity and emotional dysregulation. Albeit well-established in neuropsychiatric research, functional near-infrared spectroscopy (fNIRS) has rarely been used to study OB and BED. Here, we investigated fNIRS-based food-specific brain signalling, its association with impulsivity and emotional dysregulation, and the temporal variability in individuals with OB with and without BED compared to an age- and sex-stratified normal weight (NW) group. Prefrontal cortex (PFC) responses were recorded in individuals with OB (*n* = 15), OB + BED (*n* = 13), and NW (*n* = 12) in a passive viewing and a response inhibition task. Impulsivity and emotional dysregulation were self-reported; anthropometrics were objectively measured. The OB and NW groups were measured twice 7 days apart. Relative to the NW group, the OB and OB + BED groups showed PFC hyporesponsivity across tasks, whereas there were few significant differences between the OB and OB + BED groups. Greater levels of impulsivity were significantly associated with stronger PFC responses, while more emotional dysregulation was significantly associated with lower PFC responses. Temporal differences were found in the left orbitofrontal cortex responses, yet in opposite directions in the OB and NW groups. This study demonstrated diminished fNIRS-based PFC responses across OB phenotypes relative to a NW group. The association between impulsivity, emotional dysregulation, and PFC hypoactivity supports the assumption that BED constitutes a specific OB phenotype.

## 1. Introduction

Obesity (OB) is defined as excessive fat accumulation, described by a body mass index (BMI, kg/m²) ≥ 30 kg/m² with a prevalence of 13% in the world’s population in 2016 [1]. OB is a central risk factor for non-communicable diseases, such as coronary heart diseases or type 2 diabetes mellitus, and closely linked to affective, attentional, and eating disorders, such as binge-eating disorder (BED; [2]). BED is characterised by recurrent binge-eating episodes in the absence of regular compensatory behaviours [2] and has a lifetime prevalence of 0.85% [3]. BED is highly associated with OB, with up to 87% of individuals with BED meeting the criteria for OB (OB + BED; [4]). Notably, the high levels of eating disorder and general psychopathology in BED with associated OB exceed those seen in individuals with OB only [5,6,7].

Impulsivity, defined as a lack of considering long-term consequences, decreased sensitivity to negative consequences, and reacting to stimuli prior to complete information processing [8], is positively correlated with BMI [9,10] in self-report [11] and behavioural measures [12,13]. Individuals with OB and OB + BED have been described by increased levels of impulsivity, such as low inhibitory control [14,15,16] and reward sensitivity [17], suggesting impulsivity as an underlying mechanism of these conditions. Crucially, individuals with OB + BED were found to have greater deficits in inhibitory control than individuals with OB alone [14], particularly in the processing of food cues [18], although evidence is still inconsistent (e.g., [19]). Beyond the impairments in cognitive domains, self-report and behavioural studies have shown that OB + BED is characterised by emotional dysfunctions, including reduced emotional awareness and regulation [18,20,21]. These OB + BED-specific emotional dysfunctions exceed those observed in individuals with OB [18].

Based on functional magnetic resonance imaging (fMRI), aberrant activity patterns in a core eating disorder network comprising a ventral “reward” pathway and a dorsal “control” pathway have been placed centre stage as possible etiological factors in excess weight gain [15,22,23]. The ventral pathway, including the orbitofrontal cortex (OFC), is involved in reward and reinforcement processes [24], whereas the dorsal pathway, including the dorsolateral prefrontal cortex (DLPFC) and the inferior frontal gyrus (IFG), is associated with cognitive control [25]. While brain activity in the OFC is enhanced in response to food relative to neutral cues in OB and OB + BED compared to normal weight (NW) controls [26,27], this pattern is more pronounced in individuals with OB + BED than in individuals with OB [27,28,29]. During cognitive tasks, individuals with OB and OB + BED had reduced IFG and DLPFC activity relative to NW controls [15,22,23,25,26]. In fact, both OFC and control network hypoactivity were associated with response inhibition impairments and attentional impulsiveness in individuals with OB and OB + BED, assessed both behaviourally during a Go/NoGo task [23] and a Stroop task [27] and via self-reporting [15,23]. Additionally, the diminished recruitment of prefrontal circuitry has been linked to emotional dysfunctions in individuals with OB and OB + BED [30,31]. BED-specific neuronal activity relative to OB might form the neural basis of the conceptualisation of BED as a distinct OB phenotype [16,31,32].

Although fMRI is the most commonly used imaging method in OB and BED, it has several drawbacks, such as limited ecological validity [33]. Functional near-infrared spectroscopy (fNIRS) is an optical imaging method measuring neuronal activity based on oxygenated and deoxygenated haemoglobin [33,34]. It offers several practical advantages over fMRI, including lower costs, versatile applicability, and higher temporal resolution, despite restricted depth and spatial resolution [33,35]. Although fNIRS has enormous potential in psychiatry research [35], it has rarely been used to study OB and eating disorders [33]. The only available study using fNIRS in individuals with OB found a decreased hemodynamic response relative to individuals with overweight (BMI ≥ 25 kg/m²) in the left frontopolar area and bilateral DLPFC during a Stroop task [36]; however, no NW group has been included. Although there is no study using fNIRS in BED available, Suda et al. (2010) reported a negative correlation between fNIRS-based left OFC activity and binge-eating scores in a sample of individuals with various eating disorders [37].

An important aspect that needs to be considered when investigating brain responses to food stimuli is the signal’s temporal stability. FNIRS-based brain signalling was found to be stable over several days in a sample with overweight during various cognitive tasks, including a Go/NoGo task [38]. Only one fMRI study [39] investigated the retest reliability of food-related brain activity in individuals with OB, showing considerable the within-subject variability of brain activity and poor retest reliability on an individual level against a good mean-level reproducibility. The stability of fMRI or fNIRS brain responses over time is the most important prerequisite for recently emerging neuromodulation treatment studies in OB and eating disorders [33].

This study aimed to investigate food-specific brain responses in individuals with OB relative to individuals with OB + BED and NW using fNIRS over the OFC, IFG, and DLPFC for the first time and to determine PFC associations with impulsivity and emotional dysregulation. Specifically, this study sought to localise brain responses during a passive viewing task and a Go/NoGo task using food pictures with a high personal valence to delineate the brain regions involved in hedonic food processing (passive viewing task) and cognitive control (Go/NoGo task). We expected enhanced OFC responses across OB phenotypes relative to the NW group and diminished OFC responses in individuals with OB relative to individuals with OB + BED (OB + BED > OB > NW). DLPFC and IFG signals were assumed to be highest in the NW group followed by the OB group, and to be lowest in the OB + BED group (NW > OB > OB + BED). We expected negative associations between PFC responses and impulsivity or emotional dysregulation. The variability of fNIRS-based brain signalling over seven days was examined, hypothesising no significant PFC brain signalling variability across groups.

## 2. Materials and Methods

### 2.1. Participants and Procedure

In total, *n* = 40 participants were included. The OB group (*n* = 15) was recruited from an in-house database and information events for a behavioural weight loss treatment program at the Obesity Outpatient Unit at Leipzig University Medical Center. The OB + BED group (*n* = 13) was recruited at the outset of the study, Near Infrared Spectroscopy Neurofeedback for Binge-Eating Disorder (NIRSBED; DRKS00014752, www.drks.de). Data acquisition for the OB + BED group was performed prior to treatment randomisation in the NIRSBED trial. The NW group (*n* = 12) was age- and sex-stratified to the OB group and was recruited from Leipzig University and the population (e.g., Internet advertisements).

The inclusion criterion regarding weight status was a BMI ≥ 30.0 kg/m² for the OB and OB + BED group and 18 ≤ BMI < 25.0 kg/m^2^ for the NW group. A total of *n* = 3 participants had a lab-measured BMI < 30.0 kg/m², but in the upper overweight range (OB, *n* = 1 with 29.5 kg/m², OB + BED, *n* = 2 with 28.1 and 27.7 kg/m²). For the sensitivity analyses, all the analyses were additionally conducted excluding these 3 individuals, revealing no differences in results. Inclusion in the OB + BED group required a diagnosis of BED according to the criteria of the Diagnostic and Statistical Manual of Mental Disorders, Fifth Edition [2]. The exclusion criteria for all groups included uncorrected visual impairment; serious physical (e.g., epilepsy), neurological (e.g., dementia), and mental (e.g., attention-deficit/hyperactivity disorder) disorders; a previous or planned bariatric surgery; ongoing psychotherapy related to eating behaviours; medication intake with substantial effects on cognitive functions, weight, or eating behaviour (unless medication was stable for at least 2 months or for at least 6 months for diabetes drugs; see Supplementary Table S1); and age below 18 years. The study was approved by the Ethics Committee of the University of Leipzig (476/17-ek) and written informed consent was obtained prior to participation.

Participants were initially screened on the phone for inclusion and exclusion criteria using the diagnostic items of the Eating Disorder Examination (EDE; [40]) and parts of the Structured Clinical Interview for the Diagnostic and Statistical Manual of Mental Disorders-IV [41]. If eligible, the participants were invited for two lab assessments with seven days in between. For both appointments, the participants were instructed to fast three hours in advance. Because the data acquisition of the OB + BED group was embedded in the NIRSBED project without a second fNIRS assessment possible, temporal variability was only analysed for the OB and NW groups.

Eating Disorder Psychopathology was assessed by the global score of the Eating Disorder Examination-Questionnaire (EDE-Q; [42]; α = 0.95). Food Cravings were assessed by the sum score of the Food Cravings Questionnaire–Trait-reduced (FCQ-T-r; [43]; α = 0.98). The Behavioural Inhibition System/Behavioural Activation System (BIS/BAS; [44]) scales were used to assess different aspects of impulsivity (BIS: α = 0.79, BAS: α = 0.82). The global score of the Difficulties in Emotion Regulation Questionnaire (DERS; [45]; α = 0.96) was used to assess emotional dysregulation.

Participants with OB and OB + BED and NW controls did not differ regarding age, sex, and education, all *p* > 0.05 (Table 1). The groups differed in eating disorder psychopathology (EDE-Q) and food cravings (FCQ-T-r), both *p* < 0.001. Both the OB and OB + BED group showed larger EDE-Q global scores than the NW group, both *p* < 0.001, but the EDE-Q global scores of the OB and OB + BED group did not differ significantly, *p* = 0.521. In contrast, the OB + BED group had higher FCQ-T-r sum scores than the OB group, *p* = 0.002, and both the OB and OB + BED group had higher FCQ-T-r scores than the NW group, both *p* < 0.003.

#### Food Stimuli Selection

Participants were administered 70 food pictures derived from the Blechert et al. food-pics database [46]. Pictures included 25 pictures of savoury meals, 22 pictures of sweet meals, 11 pictures of salty meals, and 12 pictures of fruits and vegetables. At the first assessment, participants rated each picture on a 10-point Likert-Scale ranging from 0 = no current craving at all to 10 = extremely high craving and answered the question whether this food was part of a binge-eating episode during the last 4 weeks (0 = no, 1 = yes). From all the rated pictures, 12 pictures were selected based on their classification as binge food and/or their craving rating (with pictures with higher craving ratings being preferably selected; see Supplementary Table S2). These individually selected food pictures served as stimuli for the subsequent functional near-infrared spectroscopy (fNIRS) paradigms for both assessments. Instead of standardising the selection of pictures across participants, this approach allowed for an individually tailored design in which each participant watched personally valent food stimuli.

### 2.2. FNIRS Recording

#### 2.2.1. Behavioural Measures

Participants performed two computerised food-specific tasks: a passive viewing task and a Go/NoGo task. During the passive viewing task [47,48], the participants passively watched individually appetitive pictures of foods (see Supplementary Table S2). The task was comprised of 5 blocks of 12 food stimuli displayed for a maximum of 5 s each (see Supplementary Table S3). To increase task engagement and comparability with the Go/NoGo task regarding the motor component, the participants were instructed to push a joystick to indicate that they had actively processed the picture (see Supplementary Table S4).

The subsequent task was designed based on a Go/NoGo paradigm [49,50] in order to evoke brain responses generally implicated in cognitive control. The participants were presented food pictures with a high personal valence on the screen, which, in 50% of the cases, carried a red frame. The participants were instructed to push a joystick away as fast as possible, but to withhold this response for red-framed pictures. A jittered fixation cross (0.5–1.5 s) served as inter-stimulus interval. The Go and NoGo conditions equally accounted for 50% of the 144 trials performed in 6 blocks of 12 pictures of each condition in a random order, with stimuli being presented for a maximum of 2.5 s for the Go and 1.0 s for the NoGo condition. The design of this task only served to localise brain regions that are recruited during a demanding task for a subsequent neurofeedback paradigm (that is reported elsewhere), as opposed to conventional analysis of behavioural Go/NoGo data.

#### 2.2.2. Data Acquisition

A 28-channel continuous-wave NIRS system from the NIRStar Software version 15.0 (NIRx Medical Technologies LLC, Berlin, Germany) was used to measure relative changes in oxygenation levels. A 8 × 12 channel probe set was placed on the participants’ PFC, with a reference point placed at Cz according to the International 10–20 system [51], with approximately 3 cm source-detector separations except for two source-detector separations placed at 4.5 cm and 5.5 cm intervals necessary to cover the DLPFC and OFC. The oxygenated haemoglobin values were sampled at 7.8125 Hz. A total of *n* = 2 individuals in the OB group had to be excluded from data analysis for the first assessment (*n* = 1 due to recording problems, *n* = 1 due to craving ratings >2 standard deviations below the mean), leaving the first assessment analysis with *n* = 13 participants in this group.

#### 2.2.3. Data Analysis

The FNIRS data were analysed with MATLAB R2018b (The MathWorks, Inc., Natick, MA, USA) using the Brain AnalyzIR Toolbox [52]. Brain activity was defined as a concentration increase (expressed in ΔμM) in oxygenated haemoglobin values. We chose oxygenated haemoglobin as the outcome measure because it has a better signal-to-noise ratio and a stronger correlation with fMRI compared to deoxygenated haemoglobin [53] and because it is more insensitive to vascular characteristics of the covered brain tissue [54]. For data preprocessing, the baseline of raw data was manually trimmed to 30 s before and after each task. Data were downsampled to 4 Hz (with the toolbox’s Resample function, which uses interpolation and a finite impulse response anti-aliasing filter for resampling the signal to the desired frequency) and converted to optical density values which were transformed into concentration changes in oxygenated and deoxygenated haemoglobin using the modified Beer–Lambert law under consideration of an age-dependent differential pathlength factor [34]—given the high age variability in the sample (22–78 years)—and a partial pathlength factor.

The toolbox’s built-in function and a MATLAB (The MathWorks, Inc., Natick, MA, USA) based toolbox (fOLD-fNIRS optodes’ location decider; [55]) were used to identify the functional brain regions of interest (ROI; see Supplementary Table S5 and Supplementary Figure S1 and Supplementary Figure S2). The IFG covered bilateral Brodmann areas (BA) 44 and 45, the DLPFC covered bilateral BA 46, and the OFC covered bilateral BAs 10 and 11. Results were reported ROI-wise.

After preprocessing, event-related concentration changes in oxygenated and deoxygenated haemoglobin values for each ROI were modelled with a canonical hemodynamic response in a general linear model (GLM) for each participant. The GLM was solved with an iterative weighted least-squares method [56], and a third-order polynomial served as a high-pass filter. Beta estimates from the first level analysis were passed to the second-level (i.e., group) analysis as dependent variables. The main effects of the fixed factors were assessed via an analysis of variance (using the toolbox’s Anova function). Linear mixed models were used to follow up significant main effects (using the toolbox’s MixedEffects function). These models were chosen to account for design imbalances (i.e., different number of participants in the group) and to account for variability in the outcome across participants. To examine group differences, group (OB, OB + BED, NW), task (passive viewing, Go/NoGo), and Group × Task were used as fixed slopes, and participant was used as a random intercept. Due to significant group differences in self-reported emotional dysregulation and impulsivity (Table 1), we included the centred BIS and DERS scores as covariates in the analyses in order to test for their additional influence on individual differences on PFC responses irrespective of group. The results for the fixed effects after controlling for these covariates were only reported if they differed from the results without the inclusion of the covariates. For the variability analysis, group (OB, NW), assessment (first assessment, second assessment), and Group × Assessment served as fixed slopes, and participant, varying with condition and assessment, served as the random intercept. Significant main effects were followed by student’s t statistics to contrast group- and task- or assessment-wise beta estimates for oxygenated haemoglobin values from the first-level analysis between groups. Multiple comparisons were controlled by the Benjamini–Hochberg false discovery rate (FDR) correction [57].

### 2.3. Statistical Analysis of Behavioural and Self-Report Data

Statistical analysis of behavioural and self-report data was performed using R (R Foundation for Statistical Computing, Vienna, Austria) 3.6.0 [58]. All the data were examined for normality and sphericity. Nonparametric tests were applied upon violation of test assumptions. All the effects were reported as significant at a two-tailed, FDR-corrected *p* < 0.05 (i.e., *q*-values for data on brain responses).

## 3. Results

### 3.1. Group Differences in Brain Responses

#### 3.1.1. IFG

**Figure 1:**
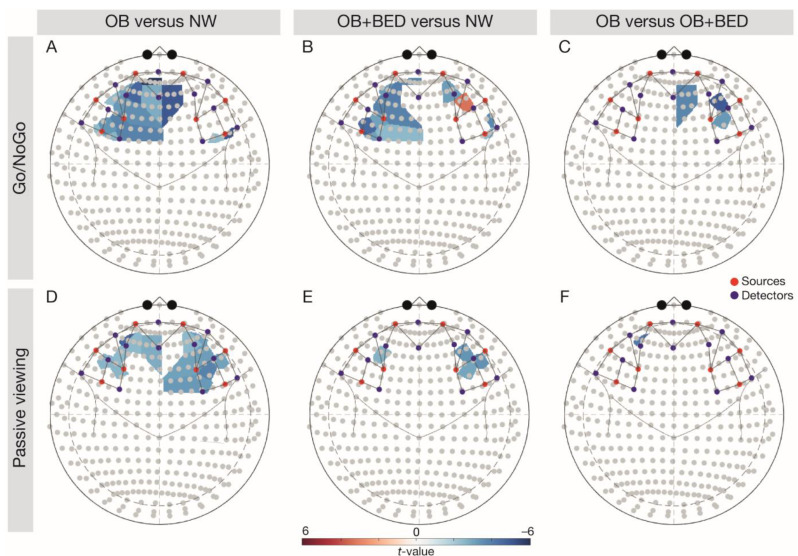
Follow-up comparisons of group- and task-wise differences in brain responses. Note: the upper row depicts the Go/NoGo task, with (**A**) OB vs. NW, (**B**) OB + BED vs. NW, and (**C**) OB vs. OB + BED. The lower row depicts the passive viewing task, with (**D**) OB vs. NW, (**E**) OB + BED vs. NW, and (**F**) OB vs. OB + BED. Red colouring indicates higher responses in the first-mentioned group, blue colouring indicates higher responses in the last-mentioned group. Red dots depict sources, blue dots depict detectors. NW, normal weight; OB, obesity; OB + BED, obesity with binge-eating disorder.

There was a significant main effect of group, *p* = 0.002, task, *p* = 0.003, and a significant interaction of Group × Task in the left IFG, *p* = 0.003 (Table 2). In line with expectations, follow-up comparisons (FDR-corrected t-tests; Figure 1) indicated higher left IFG signalling in the NW relative to the OB group during both tasks (Go/NoGo task, *t*(68) = −3.79, *p* = 0.003, passive viewing task, *t*(68) = −2.77, *p* = 0.012), and relative to the BED group during the Go/NoGo task, *t*(68) = −3.20, *p* = 0.010. A significant main effect for task was observed in the right IFG, *p* = 0.048, indicating higher brain activity during the Go/NoGo relative to the passive viewing task.

#### 3.1.2. DLPFC

A significant main effect of group was found in the right DLPFC, *p* = 0.019 (Table 2). Confirming our hypotheses, follow-up comparisons indicated significantly higher right DLPFC signalling across tasks in the NW group relative to individuals with OB, *t*(68) = −4.39, *p* < 0.001, but also in the BED group relative to individuals with OB, *t*(68) = −3.26, *p* = 0.005. There was a significant main effect of task in the left DLPFC, *p* = 0.031, reflecting higher responses during the Go/NoGo relative to the passive viewing task.

### 3.2. Effects of Impulsivity and Emotional Dysregulation

#### 3.2.1. IFG

**Figure 2:**
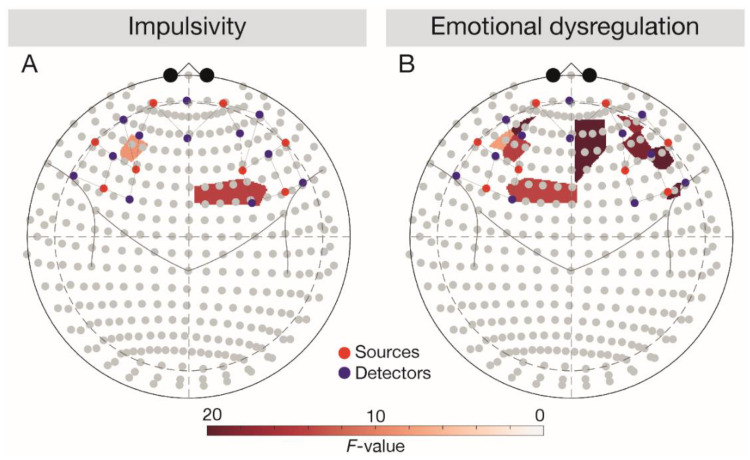
Main effects of impulsivity and emotional dysregulation depicted in 10–20 system. Results were analysed across tasks. Red dots depict sources, blue dots depict detectors. (**A**) Depicting the main effect of DERS score, (**B**) depicting the main effect of BIS score. BIS, Behavioural Inhibition System; DERS, Difficulties in Emotion Regulation Scale.

In line with expectations (Figure 2), a negative main effect of emotional dysregulation (DERS score) was observed in the left IFG (*F*(1, 3696) = 6.28, *p* = 0.043). After controlling for BIS and DERS scores, the main effect of task vanished (*F*(1, 3696) = 2.53, *p* = 0.129).

#### 3.2.2. DLPFC

Corroborating our hypotheses, we observed a positive main effect of impulsivity (BIS score) in the left DLPFC (*F*(1, 3696) = 4.99, *p* = 0.038) and a negative main effect of emotional dysregulation (DERS score) in the bilateral DLPFC (left DLPFC, *F*(1, 3696) = 10.25, *p* = 0.006, right DLPFC, *F*(1, 3696) = 7.76, *p* = 0.010). After controlling for BIS and DERS scores, a significant Task × Group interaction emerged in the bilateral DLPFC (left DLPFC, *F*(1, 3696) = 11.59, *p* = 0.002, right DLPFC, *F*(1, 3696) = 5.01, *p* = 0.043). Follow-up comparisons corroborated lower bilateral responses in the OB relative to the NW group across tasks (all *t*(66) < −2.46, all *p* < 0.049) and indicated lower signalling in the BED group relative to the NW group during the Go/NoGo task in the bilateral DLPFC (left DLPFC, *t*(66) = −3.20, *p* = 0.012, right DLPFC, *t*(66) = 3.38, *p* = 0.005). Against expectations, the follow-up comparisons also showed higher responses in the BED group relative to the OB group during the Go/NoGo task in the right DLPFC (*t*(66) = −5.41, *p* < 0.001).

#### 3.2.3. OFC

Contrasting the hypotheses, a negative main effect of emotional dysregulation (DERS score) was observed in the right OFC (*F*(1, 3696) = 10.21, *p* = 0.008). The interaction Task × Group in the right OFC turned out to be significant after controlling for BIS and DERS scores (*F*(1, 3696) = 5.72, *p* = 0.040), while the main effects of group (*F*(1, 3696) = 4.64, *p* = 0.063) and task (*F*(1, 3696) = 2.92, *p* = 0.123) vanished. Follow-up comparisons confirmed higher OFC responses in the NW relative to the OB group across tasks (Go/NoGo task: *t*(66) = −2.71, *p* = 0.026, passive viewing task: *t*(66)= −2.96, *p* = 0.026) and revealed higher right OFC responses in the BED group relative to the OB group during the passive viewing task (t(66) = -2.81, *p* = 0.034).

### 3.3. Temporal Variability in Brain Responses

**Figure 3:**
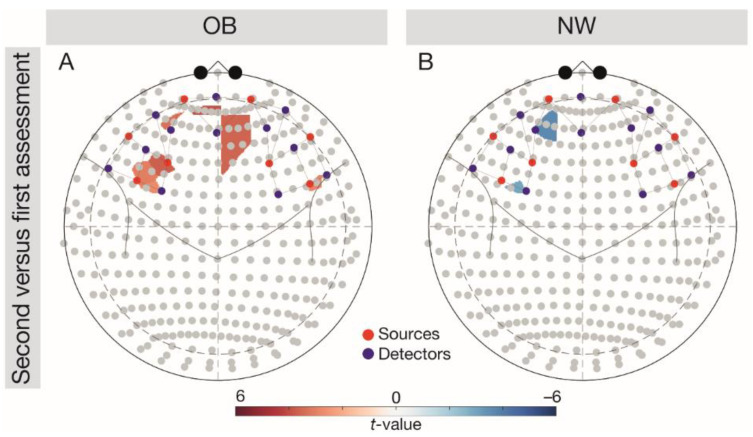
Follow-up comparisons (FDR-corrected *t*-tests) on the group-wise variability of brain responses (second-first assessment) depicted in the 10–20 system. Results were analysed across tasks. (**A**) Depicting the difference for the OB group, (**B**) difference for the NW group. Red colouring indicates higher brain responses at the second versus first, blue colouring indicates higher brain responses at the first versus second assessment. Red dots depict sources, blue dots depict detectors. NW, normal weight; OB, obesity.

We observed a significant interaction Group × Assessment in the left OFC, *F*(1, 5376) = 3.92, *p* = 0.002. Follow-up comparisons (Figure 3) indicated significantly higher left OFC responses in the NW group during the first versus second assessment (*t*(96) = −3.01, *p* = 0.007), but significantly higher brain responses in the OB group during the second versus first assessment (*t*(96) = 3.05, *p* = 0.007).

## 4. Discussion

For the first time, this study combined hypotheses from cognitive-emotional and clinical psychology and neuroscience to compare inhibitory control and hedonic processing of foods with high personal valence using fNIRS in OB phenotypes with and without associated BED and an age- and sex-stratified NW group. As hypothesised and confirming previous research, we found a consistent impairment in IFG and DLPFC functionality in the OB phenotypes compared to the NW group. There were only few inconsistent differences in the PFC responses between the OB phenotypes. Critically, this study revealed neural correlates of impulsivity and emotional dysregulation that are related to PFC hypofunctionality. Thus, our study offers evidence in favour of OB + BED as a divergent manifestation of OB characterised by specific impairments in emotional regulation and impulsivity that are underpinned by perturbations in coinciding food-specific cognitive control and emotion regulation circuits [25,30] with the PFC at their core. Regarding variability, only responses in the left OFC were not reproducible either in the OB or in the NW group, indicating that this area may be particularly susceptible for within-subject variability in perceptions of appetitive food cues.

### 4.1. Group Comparisons

As hypothesised, we found a reduction in PFC signalling across the OB phenotypes compared to individuals with NW. This is in line with previous fNIRS [36,37] and fMRI research [22,25] highlighting the PFC’s role in dietary self-regulation. Yet, the causal pathways for this well-established association between PFC perturbations and OB development are far from understood [22]. Contrasting our hypotheses and previous research demonstrating pronounced reward sensitivity in the OB + BED phenotype, we did not find distinct neural profiles in OB + BED and OB [16,32]. The diminished right OFC responses in the OB versus OB + BED group during the passive viewing task could reflect an attentional bias towards personally valent foods in OB + BED [31,59]. Importantly though, given the relationship between food-specific reward sensitivity and reduced cortico-striatal processing [27,28,29] in OB + BED, differences between OB phenotypes may only emerge in subcortical regions strongly related to reward processing.

### 4.2. Association between Brain Signalling, Impulsivity, and Emotional Dysregulation

Against the hypotheses and fMRI evidence [23,31], across groups, we found a positive association between impulsivity and food-specific left DLPFC responses. Importantly though, only individuals with OB + BED self-reported higher impulsivity relative to individuals with NW (Table 1). PFC hyperresponsivity may provide an explanation for an enhanced effort to implement food-specific self-control, despite the lack of success, in OB + BED [16,32], as mirrored by a loss of control eating during binge-eating episodes.

This study uniquely demonstrated an association between emotional dysregulation and PFC signalling, accompanying PFC hypoactivity across OB phenotypes relative to the NW group. In light of significantly larger self-reported emotional dysregulation in OB + BED (Table 1), a diminished recruitment of the DLPFC in tandem with difficulties in emotion regulation may translate into an inability to select and maintain an emotion regulation strategy to deal with negative emotions [30]. This view is consistent with a previous study reporting a negative correlation between ventromedial PFC activity and emotional dysregulation and reduced ventromedial PFC activity during the cognitive reappraisal of negative emotions in individuals with OB versus NW [30]. Reduced DLPFC activity has also been described in women with bulimia nervosa versus healthy controls during the processing of negative self-related words [60].

### 4.3. Variability of Brain Signalling in the OB and NW Groups

Partially confirming our hypothesis that brain signalling would not vary over seven days in the OB and NW group, we found no significant differences between assessments in brain responses in all our ROIs except for significant variability in left OFC activity. Large variability of individuals with OB has previously been described in fMRI-based brain activity [39] and in behavioural tasks [14,19], with inter-subject variability in task performance being closely related with inter-subject variability in brain activity [61]. We speculate that neural variability may parallel task performance in the Go/NoGo task (see Supplementary Table S4).

### 4.4. Limitations

Due to the cross-sectional nature of the study, determining the causal pathways underlying the association between OB and PFC hypoactivity is a viable route for future prospective studies. Given our moderately sized sample of *n* = 40 individuals for the analysis of group differences, studies with larger samples are required to replicate the present findings. The inclusion of *n* = 3 individuals in the upper overweight range may have led to underestimated differences in brain responses between OB phenotypes and may limit the generalisability to OB. Likewise, we did not systematically select participants according to their comorbidities (see Table 1) or medication (see Supplementary Table S1) and can thus not exclude that our results were affected by associated diseases and medication intake. Finally, the relatively low spatial resolution of fNIRS potentially precludes inferences on closely located brain regions, such as the OFC and the DLPFC. Related to this, some channels were not specifically assigned to only one BA (see Supplementary Table S5), thereby limiting ROI-specific interpretations.

## 5. Conclusions

Overall, this study takes an important step towards a better understanding of the neural, cognitive, and emotional correlates of distinct OB phenotypes as compared to NW individuals. To the best of our knowledge, this is the first study that applied fNIRS in individuals with OB, OB + BED, and NW using personally valent stimuli and validated behavioural tasks. The major strengths of this study include the use of objective anthropometrics, BED diagnosis based on clinical interview, and the careful group stratification.

Our results suggest that differences in cognitive (i.e., impulsivity) and emotional (i.e., emotional dysregulation) traits that may contribute to binge-eating behaviours are mirrored in aberrant PFC responses. Consequently, inhibitory and emotion regulatory skills should be placed centre stage in the treatment of BED to enhance food-related self-regulation skills. These findings pave the way towards the development of brain-based intervention strategies [33] with the DLPFC as a candidate target area. Indeed, preliminary studies provide favourable evidence for neuromodulation techniques to serve as an adjunct treatment for OB and OB + BED [62]. Considering its numerous advantages, including easy applicability and clinical utility, our findings opened the window to the application of fNIRS to better understand disorder trajectories in order to develop appropriate treatments [35]. Promising findings from attention-deficit/hyperactivity disorder [63] have provided the impetus for pioneering research on the effects of fNIRS neurofeedback training in individuals with BED (DRKS00014752, www.drks.de). In conclusion, we advocate for more research on the synergistic effects of neuromodulation techniques in tandem with well-established treatments in the context of OB and OB + BED, using methods easily applicable in clinical practice.

## Figures and Tables

**Table 1:** Participants’ sociodemographic and clinical characteristics at the first assessment.

	OB *n* = 15	OB + BED *n* = 13	NW *n* = 12	Test Statistics	Effect Size	*p* Value	Post-Hoc Tests
	*M (SD)*	*M (SD)*	*M (SD)*				
Age, years	50.07 (17.64)	42.71 (12.77)	56.42 (18.66)	*F*(2, 37) = 1.88	η² = 0.09	0.170	
Sex, female: *n* (%)	9 (60%)	11 (79%)	8 (67%)		*V* = 0.16	0.386	
Education: *n* (%)					*V* = 0.23	0.116	
<12 years	11 (74%)	7 (54%)	4 (33%)				
≥12 years	4 (27%)	6 (46%)	8 (67%)				
Body mass index, kg/m²	39.23 (7.52)	35.13 (5.24)	23.60 (2.03)	*F*(2, 37) = 28.14	η² = 0.60	<0.001	OB, OB + BED > NW
Weight status: *n* (%)					*V* = 0.71	<0.001	OB, OB + BED > NW
Obesity (BMI ≥ 30 kg/m²)	14 (93%)	11 (85%)	0				
Overweight (BMI ≥ 25 kg/m²)	1 (7%)	2 (15%)	0				
Normal weight (BMI < 25 kg/m²)	0	0	12 (100%)				
Number of participants with comorbidities	10 (67%)	6 (46%)	4 (33%)	Χ² (2, *N* = 40) = 2.8	*V* = 0.26	0.247	
Comorbidities: *n* (%)					*V* = 0.26	0.306	
Hypercholesterolemia	2 (13%)	0	1 (8%)				
Hypertension	6 (40%)	3 (23%)	4 (33%)				
Diabetes mellitus (Type I or II)	4 (29%)	0	0				
Thyroid diseases	1 (7%)	2 (15%)	0				
Pulmonary diseases	1 (7%)	2 (15%)	0				
Other	1 (7%)	2 (15%)	0				
EDE-Q global score	2.44 (1.36)	2.93 (1.02)	0.64 (0.39)	*F*(2, 34.41) = 20.70	η² = 0.47	<0.001	OB, OB + BED > NW
FCQ-T-r sum score	34.46 (14.61)	57.21 (13.63)	19.17 (4.69)	*H*(2) = 24.30	η² = 0.60	<0.001	OB + BED > OB > NW
BIS/BAS, BIS mean score	2.95 (0.52)	3.14 (0.57)	2.63 (0.57)	*F*(2, 37) = 3.81	η² = 0.17	0.031	OB + BED > NW
BIS/BAS, BAS mean score	3.13 (0.51)	2.80 (0.28)	3.00 (0.38)	*F*(2, 37) = 2.60	η² = 0.12	0.086	
DERS global score	76.33 (22.48)	98.33 (31.30)	58.58 (9.74)	*H*(2) = 17.31	η² = 0.42	<0.001	OB + BED > OB > NW

Note: BAS, Behavioural Activation System; BIS, Behavioural Inhibition System; BMI, Body mass index; DERS, Difficulties in Emotion Regulation Scale; EDE-Q: Eating Disorder Examination-Questionnaire; FCQ-T-r, Food Cravings Questionnaire-Trait-reduced; NW, Normal Weight; OB, Obesity; OB + BED, Obesity and Binge-Eating Disorder. Effect sizes for continuous variables were reported as η² and interpreted as small (0.01), medium (0.06), and large (0.14). Effect sizes for categorical variables were reported as Cramer’s V and interpreted as small (0.1), medium (0.3), and large (0.5).

**Table 2:** Main effects of task, group, and the interaction on the fNIRS-based PFC responses.

ROI		Contrast	Test Statistics	Uncorrected *p*-Value	FDR-Corrected *p*-Value
IFG	left	task	*F*(1, 3808) = 9.90	0.002	0.003
		group	*F*(1, 3808) = 9.17	0.003	0.002
		Task × Group	*F*(1, 3808) = 10.2	0.001	0.003
IFG	right	task	*F*(1, 3808) = 6.90	0.009	0.048
		group	*F*(1, 3808) = 5.28	0.022	0.058
		Task × Group	*F*(1, 3808) = 3.38	0.066	0.079
DLPFC	left	task	*F*(1, 3808) = 7.09	0.008	0.031
		group	*F*(1, 3808) = 3.92	0.048	0.077
		Task × Group	*F*(1, 3808) = 1.02	0.313	0.313
DLPFC	right	task	*F*(1, 3808) = 0.08	0.778	0.778
		group	*F*(1, 3808) = 7.25	0.007	0.018
		Task × Group	*F*(1, 3808) = 2.65	0.104	0.139
OFC	left	task	*F*(1, 3808) = 1.90	0.168	0.223
		group	*F*(1, 3808) = 0.86	0.354	0.405
		Task × Group	*F*(1, 3808) = 3.11	0.078	0.156
OFC	right	task	*F*(1, 3808) = 3.17	0.075	0.086
		group	*F*(1, 3808) = 2.39	0.122	0.122
		Task × Group	*F*(1, 3808) = 4.40	0.036	0.058

Note. DLPFC, dorsolateral prefrontal cortex; FDR, False Discovery Rate; IFG, inferior frontal gyrus; OFC, orbitofrontal cortex.

## Data Availability

The data presented in this study are available on request from the corresponding author. The data are not publicly available due to the sensitivity of the data (both clinical and neuroimaging data may allow inferences to individual persons.

## Supplementary Materials

The following are available online at https://www.mdpi.com/2076-3425/11/1/19/s1: Table S1: Participants’ medication at the first assessment; Table S2: Participants’ ratings of food stimuli and nutritional information of food stimuli; Table S3: Watching time and number of pictures when a joystick was pushed prior to expiration time in the passive viewing task; Ta-ble S4: Group- and assessment-wise number of commission errors and go reaction time in the Go/NoGo task; Table S5: Assignment of source-detector pairs to brain areas; Figure S1: Sensitivity profile of the montage; Figure S2: The position of the three ROIs DLPFC, IFG, and OFC in Colin27 atlas.
