# Supplementary material for: Evidence of fNIRS-Based Prefrontal Cortex Hypoactivity in Obesity and Binge-Eating Disorder
Source: Brain Sci. 2020 Dec 26;11(1):19. doi: 10.3390/brainsci11010019 (PMC7823505; doi:10.3390/brainsci11010019)
Supplement: Supplementary file 1 [file brainsci-11-00019-s001.pdf]

Supplementary Material for:

Sarah A. Rösch, Ricarda Schmidt, Michael Lührs, Ann-Christine Ehlis, Swen Hesse, Anja Hilbert.

Evidence of fNIRS-Based Prefrontal Cortex Hypoactivity in Obesity and Binge-Eating Disorder

Supplementary Text

1. Supplementary Methods

2. Supplementary Results

Supplementary Tables

Supplementary Table S1. Participants' medication at the first assessment

Supplementary Table S2. Participants' ratings of food stimuli and nutritional information of food stimuli

Supplementary Table S3. Watching time and number of pictures when a joystick was pushed prior to expiration time in the passive viewing task

Supplementary Table S4. Group- and assessment-wise number of commission errors and go reaction time in the Go/NoGo task

Supplementary Table S5. Assignment of source-detector pairs to brain areas

Supplementary Figures

Supplementary Figure S1. Sensitivity profile of the montage

Supplementary Figure S2. The position of the three ROIs DLPFC, IFG, and OFC in Colin27 atlas

Supplementary References

Brain Sci. **2021**, *11*, 19. https://dx.doi.org/10.3390/brainsci11010019

1. Supplementary Methods

1.1. Participants' medication

Supplementary Table S1. Participants' medication at the first assessment

	**OB** *n* = 15	**OB+BED** *n* = 13	**NW** *n* = 12	**Test statistics**	**Effect size**	***p* value**
Number of participants with medication	10 (67%)	6 (46%)	4 (33%)	X^2 (2, *N* = 40) = 2.8	*V* = .26	.247
Medication: *n* (%)					*V* = .62	.334
Cholesterol and blood lipid lowering drugs	2 (13%)	0	1 (8%)			
Antihypertensive drugs	6 (40%)	3 (23%)	4 (33%)			
Diabetes drugs	4 (29%)	0	0			
Thyroid drugs	1 (7%)	2 (15%)	0			
Pulmonary drug	0	2 (15%)	0			
Psychotropic drugs	1 (7%)	2 (23%)	0			
Gout drugs	0	1 (8%)	0			
Cardiac drugs	0	1 (8%)	0			
Cortisone drugs	1 (7%)	0	0			
Opioids	1 (7%)	0	0			
Gastroesophageal reflux disease drugs	1 (7%)	0	0			
Malaria prophylaxis	1 (7%)	0	0			
Anti-inflammatory drugs	1 (7%)	0	0			
Johannis herbs	0	1 (8%)	0			
Muscoskeletal medication	0	1 (8%)	0			

The number of participants who took medication stable according to our inclusion criteria did not differ between groups, X^2 (2, *N* = 40) = 2.8, *p* = .247 (see Supplementary Table S1). Likewise, medication was not differentially distributed between groups (Fisher's exact test, *p* = .334).

1.2. Food Stimuli Ratings

There was no significant main effect of group, $F(2, 37) = 0.07$, $p = .937$, $\eta^2 = .00$, in craving ratings. In contrast, there was a statistically significant, large difference in the proportion of food pictures being classified as binge food, $H(2) = 23.48$, $p < .001$, $\eta^2 = .42$. Pairwise Bonferroni-corrected comparisons for the proportion of food pictures being classified as binge food validated the group assignment: there were significant differences between the group with obesity and binge-eating disorder (OB+BED) compared to the group with obesity (OB), $p = .001$ and the group with normal weight (NW), $p < .001$, but not between OB and NW groups, $p = .163$. Based on the nutritional information provided by Blechert et al. (2014) [1], there was no statistically significant difference in caloric content of the selected food stimuli between groups, $H(2) = 4.16$, $p = 0.125$, $\eta^2 = 0.06$. Participants' ratings as well as nutritional information are shown in Supplementary Table S2.

Supplementary Table S2. Participants' ratings of food stimuli and nutritional information of food stimuli

	OB<br>*n* = 15	OB+BED<br>*n* = 13	NW<br>*n* = 12	Test statistics	Effect size	*p* value	Post-hoc tests
	M (SD)	*M (SD)*	*M (SD)*				
Craving	65.72 (25.99)	63.36 (25.99)	63.60 (21.81)	$F(2, 37) = 0.07$	$\eta^2 = .000$	.937	
Binge food, *n* (%)	4.20 (35%)	11.95 (96%)	0.33 (3%)	$H(2) = 23.48$	$\eta^2 = .422$	< .001	OB+BED > OB,<br>OB+BED > NW
Kilocalories/100g	565.65 (277.22)	786.42 (261.28)	575.76 (362.92)	$H(2) = 4.16$	$\eta^2 = .060$	.125	

Note. The ratings for craving ranged from 0 to 100, with higher values indicating higher levels of craving. Binge food describes the group-wise mean number of stimuli displayed during the tasks that were classified as binge foods (in total, 12 food stimuli were displayed). Information on caloric content derived from [1]. Effect sizes were reported as η^2 and interpreted as small (.01), medium (.06), and large (.14).

1.3. FNIRS Data Acquisition

The probabilistic path of photon through cortex were estimated using the Monte-Carlo transport software tMCimg via the Atlas Viewer from Homer2 [2,3]. The optodes' placement and the results of the simulation are shown in Supplementary Figure S1. Before starting the experiment a calibration was performed in order to check each optode's signal quality.

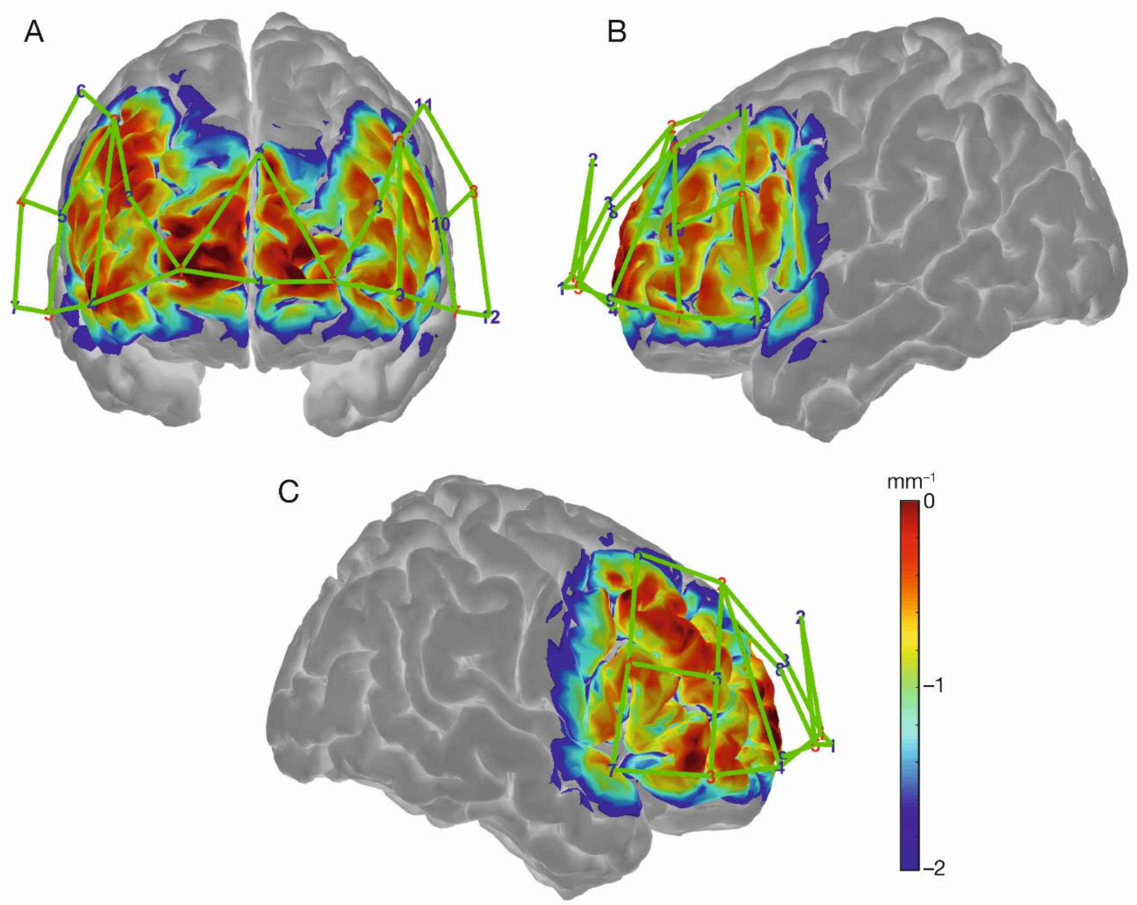

Supplementary Figure S1. Sensitivity profiles for cortical regions of interest. Color scale depicts relative sensitivity to hypothetical cortical activation logarithmically from -2 to 0 in log10 units.

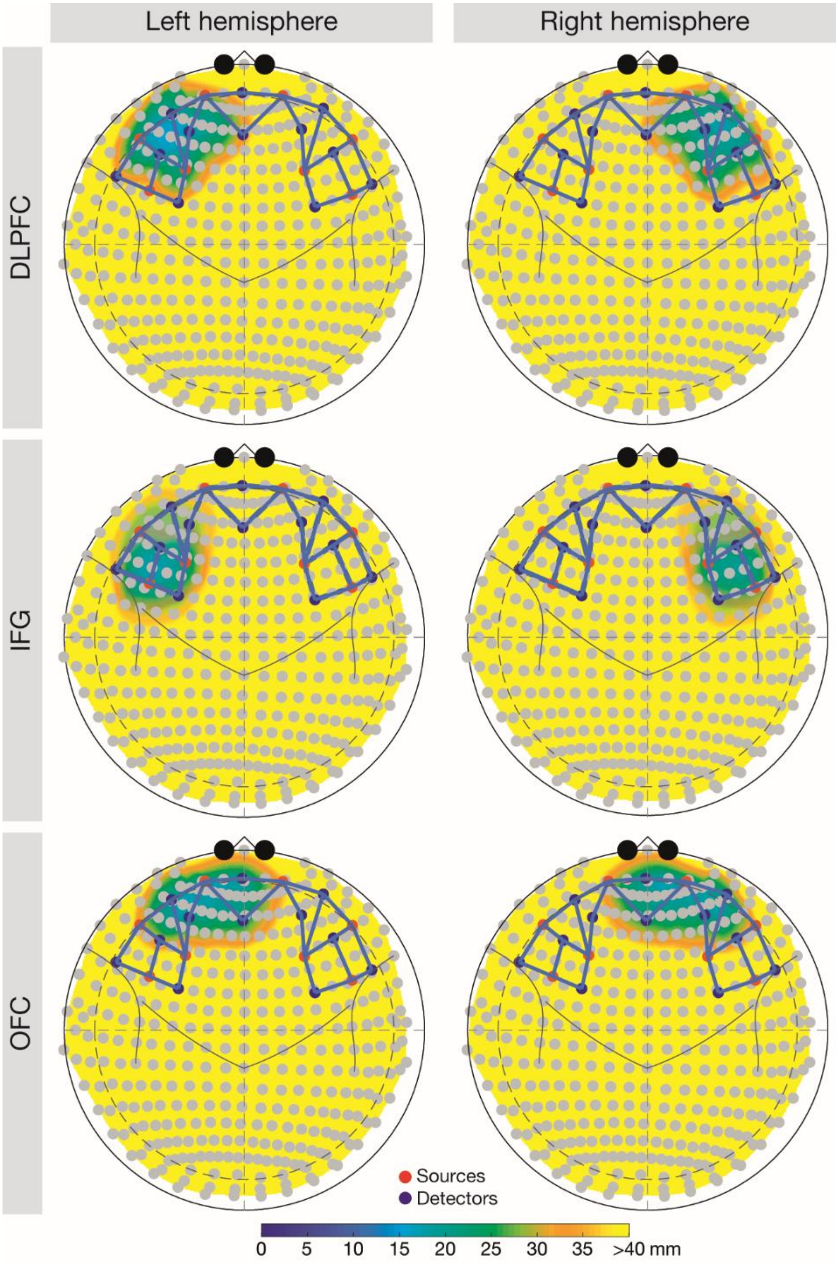

Supplementary Figure S2. The position of the three ROIs DLPFC (Brodmann area [BA] 46), IFG (BA 44 and 45) and OFC (BA 10) in Colin27 [4] atlas, which was used in combination with the automatic anatomical labeling toolbox. The color map represents the depth from each source or detector in the ROI to the head surface in topology maps (Clarke azimuthal map projection). Yellow colour indicates a depth of greater than 40mm, which is inaccessible to fNIRS light. DLPFC, dorsolateral prefrontal cortex; IFG, inferior frontal gyrus; OFC, orbitofrontal cortex

Supplementary Table S3. Assignment of source-detector pairs to brain areas

Channel	Brodmann Area	Description	fOLD area	ROI assignment in the study
S1 – D1	BA 10 right	Frontal superior right	BA 10/11 right	OFC right
S1 – D2[a]	BA 10 right	Frontal superior right	BA 10 right	OFC right
S1 – D3	BA 10 right	Frontal middle right	BA 10 right	OFC right
S1 – D4	BA 10 right	Frontal middle right	BA 10/11 right	OFC right
S2 – D3	BA 46 right	Frontal middle right	BA 9/45/46 right	DLPFC right
S2 – D4[b]	BA 46 right	Frontal middle right	BA 46 right	DLPFC right
S2 – D5	BA 45 right	Frontal inferior triangularis right	BA 45/46 right	IFG right
S2 – D6	BA 44 right	Frontal middle right	BA 9/44/45 right	IFG right
S3 – D4	BA 47 right	Frontal inferior orbital right	BA 45 right	IFG right
S3 – D5	BA 45 right	Frontal inferior triangularis right	BA 45 right	IFG right
S3 – D7	BA 38 right	Temporal pole middle right	none	excluded
S4 – D5	BA 45 right	Frontal inferior triangularis right	BA 45	IFG
S4 – D6	BA 6 right	Precentral right	BA 44 right	excluded
S4 – D7	BA 38 right	Temporal pole superior right	None	excluded
S5 – D1	BA 10 left	Frontal superior medial left	BA 10/11 left	OFC left
S5 – D2[a]	BA 10 left	Frontal superior left	BA 10 left	OFC left
S5 – D8	BA 10 left	Frontal superior left	BA 10/46 left	OFC left
S5 – D9	BA 11 left	Frontal superior left	BA 10/11 left	OFC left
S6 – D8	BA 46 left	Frontal middle left	BA 9/45/46	DLPFC left
S6 – D9[b]	BA 46 left	Frontal middle left	BA 46 left	DLPFC left
S6 – D10	BA 45 left	Frontal middle left	BA 45/46 left	IFG left
S6 – D11	BA 44 left	Frontal middle left	BA 9 left	IFG left
S7 – D9	BA 46 left	Frontal middle left	BA 45 left	IFG left

Channel	Brodmann Area	Description	fOLD area	ROI assignment in the study
S7 – D10	BA 45 left	Frontal inferior triangularis left	BA 45 left	IFG left
S7 – D12	BA 47 left	Frontal inferior orbital left	None	excluded
S8 – D10	BA 45 left	Frontal inferior triangularis left	BA 44/45 left	IFG left
S8 – D11	BA 6 left	Precentral left	BA 44 left	excluded
S8 – D12	BA 6 left	Frontal inferior oper left	None	excluded

Note. BA, Brodmann area; DLPFC, dorsolateral prefrontal cortex; fOLD, fNIRS Optodes' Location Decider; IFG, inferior frontal gyrus; OFC, orbitofrontal cortex. The description in columns 2 and 3 is based on the BrainAnalyzIR [5] toolbox, the description in Columns 4 and 5 is based on fNIRS Optodes' Location Decider [6] with at least 30% specificity for the corresponding BA.
[a] source-detector separation at 4.5 cm. [b] source-detector separation at 5.5 cm.

2. Supplementary Results

2.1. Behavioural data - passive viewing task

Supplementary Table S4. Watching time and number of pictures when a joystick was pushed prior to expiration time in the passive viewing task

	Assessment	OB[a] n = 10	OB+BED n = 12	NW n = 12	Test statistics	p-value	Effect size	Post-hoc tests
		M (SD)	*M (SD)*	*M (SD)*				
Watching time per picture, s	T1	1.80 (1.32)	1.33 (0.78)	2.27 (1.39)	$H(2) = 226.54$	< .001	$\eta^2 = .11$	OB+BED < OB < NW
	T2	1.81 (1.26)		1.73 (0.99)	$H(2) = 1.19$	.274	$\eta^2 = .00$	
Pictures pushed prior to expiration time, n (%)	T1	54.30 (91%)	59.33 (99%)	52.42 (87%)	$H(2) = 2.60$	.272	$\eta^2 = .05$	
	T2	54.36 (91%)		58.75 (97%)	$H(1) = 0.262$	.608	$\eta^2 = .00$	OB < NW

Note. Data are only displayed for individuals with valid fNIRS data. Pictures pushed describes the group-wise mean number of stimuli displayed during the tasks where the joystick has been pushed before the maximum viewing time had been expired (in total, 60 food stimuli were displayed). Effect sizes were reported as η^2 and interpreted as small (.01), medium (.06), and large (.14).

[a]Due to recording problems, data for the passive viewing task were not available for $n = 3$ individuals in the first and for $n = 2$ individuals in the second assessment for the OB group.

For the first assessment, there was a statistically significant, medium-sized difference in the time participants observed pictures in the passive viewing task, $H(2) = 226.54$, $p < .001$, $\eta^2 = .11$ (Supplementary Table S4). The shortest viewing times were observed in the OB+BED group, followed by the OB group, with the NW group showing the largest viewing times. Pairwise Bonferroni-corrected comparisons confirmed significant differences between all groups, all $p < .001$. For the second assessment, there was no statistically significant difference in the time participants in the OB and NW groups observed pictures in the passive viewing task, $H(1) = 1.20$, $p = .274$, $\eta^2 = .00$.

Likewise, there was no statistically significant in either of the assessments in the number of pictures where the joystick was pushed prior to expiration time, first assessment: $H(2) = 2.60$, p = .272, $\eta^2 = .05$, second assessment: $H(1) = 0.26$, p = .609, $\eta^2 = .00$.

2.2. Behavioural data - Go/NoGo task

Supplementary Table S5. Group- and assessment-wise number of commission errors and go reaction time in the Go/NoGo

	Assessment	OB[a] n = 13	OB+BED n = 12	NW n = 12	Test statistics	p-value	Effect size	Post-hoc tests
		M (SD)	*M (SD)*	*M (SD)*				
Commission errors, n	T1	1.15 (1.52)	1.08 (1.08)	1.00 (1.41)	$H(2) = 0.23$	.890	$\eta^2 = .05$	
	T2	1.33 (1.50)		0.25 (0.62)	$H(1) = 5.34$	.021	$\eta^2 = .17$	OB > NW
Reaction time, ms	T1	2.19 (0.46)	3.86 (0.51)	0.74 (0.43)	$H(2) = 2361.20$	< .001	$\eta^2 = .89$	OB+BED > OB > NW,
	T2	2.32 (0.50)		0.76 (0.54)	$H(1) = 1396.90$	< .001	$\eta^2 = .72$	OB > NW

Note. Data are only displayed for individuals with valid fNIRS data. Effect sizes were reported as η^2 and interpreted as small (.01), medium (.06), and large (.14).
[a]n = 12 individuals for the second assessment

At the first assessment, groups did not differ in the number of commission errors, $H(2) = 0.23$, $p = 0.890$, $\eta^2 = .05$ (Supplementary Table S5). However, there was a statistically significant, large difference in reaction time, $H(24) = 2361.20$, $p < .001$, $\eta^2 = .89$, with the largest reaction times being observed in OB+BED, followed by the OB group, and the NW group showing the shortest reaction times. Pairwise Bonferroni-corrected comparisons confirmed significant differences between all groups, all $p < .001$.

Considering the second assessment only, the OB group showed a significantly higher number of commission errors than individuals with NW, $H(2) = 5.34$, $p = .021$, $\eta^2 = .17$, and there was a statistically significant, large difference in reaction time, with individuals with OB having larger reaction times as compared to individuals with NW, $H(23) = 1396.90$, $p < .001$, $\eta^2 = .72$.

Supplementary References

1. Blechert, J.; Meule, A.; Busch, N.A.; Ohla, K. Food-pics: An image database for experimental research on eating and appetite. *Front Psychol* **2014** *5*,1–10.
2. Aasted, C.M.; Yücel, M.A.; Cooper, R.J. Anatomical guidance for functional near-infrared spectroscopy: Atlasviewer tutorial. *Neurophotonics* **2015** *2*,020801–020801.
3. Boas, D.A.; Culver, J.P.; Stott, J.J.; Dunn, A.K. Three dimensional monte carlo code for photon migration through complex heterogeneous media including the adult human head. *Opt Express* **2002** *10*,159-170.
4. Holmes, C.J.; Hoge, R.; Collins, L.; Woods, R.; Toga, A.W.; Evans, A.C. Enhancement of MR images using registration for signal averaging. *J Comput Assist Tomogr* **1998** *22*, 324–333.
5. Santosa, H.; Zhai, X.; Fishburn, F.; Huppert, T. The NIRS Brain AnalyzIR Toolbox. *Algorithms* **2018** 11:73.
6. Zimeo Morais, G.A.; Balardin, J.B.; Sato, J.R. 2018. FNIRS Optodes' Location Decider (fOLD): a toolbox for probe arrangement guided by brain regions-of-interest. *Scientific Reports* **2018** *8*:1–11.
